# Many Items

**Published:** 1890-09

**Authors:** 


**Shock in Heart Disease.**—A Jew having failed to sell a coat at $20, and at $15, took his customer away back in the back part of the store, behind the stair steps, and in a hoarse whisper behind his hand, said, "You shoost take dat coat along mit you for twelve tollars."

"All right" says the customer, "but what is the meaning of all this mystery? why couldn't you tell me that out front?"

"Sh—!" said the Jew with a look of terror on his face, "You see dat schmall leedle man mit de front door? dat was mine leedle brudder; he got heart disease, and so help me goodness gracious, if he hear me say twelve tollars for dat coat, he shoost drop down dead mit his tracks!"

THE MEDICAL PROFESSION OF LOUISIANA has again failed with the State legislature in the matter of alaw to regulate practice. The scheme was defeated by *eight homeopaths*, it seems; and since we learn that the ubiquitous C. E. Fisher who left these parts between two suns, and was supposed to be in California, has turned up in New Orleans, we think we can see his "fine Italian hand" in the nefarious work. Of course the petition was defeated by gross misrepresentation and the now thread-bare. pretext that it is a "war on homeopathy," war on moonshine. No such entity exists as a homeopathic physician; that is long since played out.

### Medical News and Miscellany

The next International Medical Congress will be held in Rome in 1893.

It is claimed that subbenzoate of bismute is almost a specific cure for soft chancre.

Married.—In Austin, September 3, 1890, Dr. W. B. Gibson to Mrs. Rosa Hughey, both of Austin.

Mr. Christopher Heath, in a recent lecture on strangulated hernia, protests strongly against the use of ice in that connection.—*Gaillard's Medical Journal.*

Death of Mrs. West.—The JOURNAL learns with deep regret just as we go to press, of the death of Mrs. West, of Galveston, wife of Dr. Hamilton A. West, of that city, Professor of Practice in the Texas Medical College. She died in child birth on the 17th inst. Dr. West has the sympathy of the entlre medical profession, with whom he is very popular.

Removals.—Dr. A. M. Pelton has removed from Deming's Bridge to Ashby, Matagorda county.

Dr. G. W. McCaleb has removed from Elm Grove to Gonzales.

Dr. W. A. McGee has removed from Alpine to Dripping Springs, Hays county.

Dr. A. D. Burrough has removed from Lovelady to Houston.

Variations in the Caustic Action of Carbolic Acid.—It is claimed on authority of Mr. Charles that carbolic acid, if dissolved in glycerine or alcohol is not caustic, whatever be the degree of concentration. An aqueous solution or even a small per cent. of water added to the alcohol or glycerine solution will act as a caustic to the skin and mucous membrane.—*Le. Bul. Med.* —*Weekly Med. Review.*

**Dr. B. F. Church**, of Austin, who was recently honored with the Presidency of Travis County Medical Society, has been appointed by President Burts, of State Medical Association, Chairman of Section on Dermatology, vice Dr. W. T. Baird, of Dallas,—under the resolution that unless a chairman of Section give notice to the General Secretary of his acceptance of the office within a given time, the chairmanship shall be declared vacant and shall be filled by appointment by the President. The leaven of reform is working.

---

**Salicylic Acid as a Prophylactic in Scarlatina.**—Continuing the reseaches of Barkes, DeRossa has administered to 66 children belonging to families in which there was scarlet fever, a daily dose of gr. iss to jvss according to age, of salicylic acid. He concludes that salicylic acid acid absolutely prevents the development of scarlatina even if be taken in time and in sufficient dose. Even when administered late in too small a dose, it renders the disease very mild and benign.—*Annals Gyn. and Ped.* —*Weekly Medical Review.*

---

**Dr. Jno. Preston**, first assistant physician at the State Lunatic Asylum, has resigned his position and will remove to San Antonio to engage in general practice. It is generally understood that Dr. Preston will be a candidate for the position of superintendent of the State Lunatic Asylum when the new administration comes in; and it is generally believed in medical circles hereabout that he will receive the appointment. His experience with that class of patients and his popularity with the medical profession constitute strong claims for the place.

---

**Small-Pox Scare.**—The occurrence of a case or two of small-pox in the interior of the State has caused a flutter of excitement at Waco and Marlin. The little town of Marlin, a few miles distant from Waco, a rival cotton market by the bye, promptly instituted "shot-gun quarantine." It required the presence of the Governor and the issuance of an executive order, to remove it; and even then, Marlin "bucked" and seemed to question whether or not "the little cavalry man," Gov. Ross, was "a bigger man than Grant." At last accounts all was quiet along the lines, and the shot gun had been lowered.

---

**Hæmaturia and Gorden Rhubarb.**—Several correspondents of the *Lancet* have recently reported some unusual urinary troubles, consequent upon eating ordinary rhubarb, or pie plant, as it is commonly called. The symptoms are frequent micturition, hæmaturia, dysuria, and lumbar pains. This effect of the rhubard seems dependent upon the use of hard water for drinking purposes, the oxalic acid of the rhubarb combing with the calcium in the water and forming numerous small crystals of oxalate of calcium that—it is suggested—lacerate the uriniferous tubules in passing through them. Similar consequences have been noted of eating gooseberries and acid apples; and an explanation of obscure urinary troubles in localities where hard water is used is thus offered.—N. Y. Med. Journal.

---

**Dr. Geo. B. Fowler,** (in Medical Record), recommends a method of artificial infant feeding which will be found satisfactory. Put four tablespoonsful of rice into three pints of water and boil half an hour, then ret on back of range to simmer during the day, water being added occasionally to maintain the original three pints. At night strain through a colander and place on ice. When cold a paste is formed. Three tablespoonsful of this paste are added to each half pint of milk and fed during the next day, a fresh supply being under headway in the mean time. Rice is astringent, so if there be constipation he uses farina, prepared in the same way and proportions. The hydrated starch granules prevent the formation of solid clots of casein. The starch thus treated is easily digested by a child even two months old. [The addition of a small quantity of chloride of sodium to the paste or milk would not only make it more palatable but easier of digestion.—Ed.]

---

**Treatment of Cystitis in Women.**—Dr. T. More Madden, of Dublin, read a paper in which he describes the method of treatment of this distressing condition that had been very successful in his hands. His plan consisted first in the full dilation of the urethral canal with an instrument exhibited, so as to paralize the contractility of the sphincter vesicæ and canal, and thus produce a temporary incontinency of urine, and, secondly, in the direct application through the same instrument of carbolic acid and glycerine to the diseased endo-vesical mucus membrane. Any pain thus caused may be prevented by the previous topical application of a solution of cocain. The proceedure recommended seldom requires to be repeated more than once or twice, and then at intervals of a week or ten days. This treatment, combined with the internal use of boric acid, rarely failed to effect a rapid cuse.—[A report from the Medical Congress at Berlin to the Medical Record.]

---

The deplorable state of medical journalism in the West is evidenced by the recent issues of two contemporaries. The first contains the account of an assault with intent to kill perpetrated on the editor, from which he escaped unscathed, (so he says) and in token of which he publishes his physiognomy, presumably after the affray, as there are no scratches on it. Being unable to ascend a dung-hill and give vent to pæns of praise, like his feathered mentors, he takes this method of informing the public he is well, and hopes for a continuance of their patronage, etc. The other has some alleged wit, capitally disguised, about "Jack the Ripper," containing allusions to L. T.———, of England, which reminds us of a toy dog yelping at the king of beasts, followed by remarks in a sour grapes strain about post-graduate schools. The lucubration winds up with some doggrel patterned after the "Rejected Addresses." And this is medical journalism. God save the mark! No wonder western men and journals are held in such low esteem by the profession in the east and in Europe.—*North American Practitioner.*

---

A $200,000 Libel Suit.—Suit has been entered by William Radam, manufacturer of Radam's Microbe Killer, against the *Druggists Circular*, of New York, for $200,000 damages, the largest amount so far as heard from that was ever asked for in a libel suit of this kind.

The pleadings show that the action is brought to recover damages claimed to have been done the business of the plaintiff by an article published in the *Druggists Circular* for September, 1889. This article gave the result of an analysis of the Microbe Killer, made by Dr. R. G. Eccles, a prominent chemist of Brooklyn, who stated that an identical preparation could be made by the following formula:

Oil of vitriol (impure)......4 drams.
Muriatic acid (impure)......1 dram.
Red wine, about...........1 ounce.
Well or spring water.......1 gallon.

This mixture, it was alleged, could be made at a cost of less than five cents per gallon, for which Radam charged three dollars.

It was further alleged that while, when properly used, sulphuric acid, the principal constituent of the Microbe Killer, was a valuable medicine, it was, when taken without due caution and advice, a slow but certain cumulative poison; and the theories advanced by Radam, as to the causes of diseases and the proper method of treatment, were alleged to be totally erroneous. Col. Robert B. Ingersoll, the famous lecturer, is the counsel for the plaintiff.

The *Druggists Circular*, which is published at 72 William str., New York, expresses a desire to hear of any case in which unfavorable results have followed the administration of the Microbe Killer, or any other fact that would be interesting under the circumstances. They claim to have published this analysis without malice, and with the sole intention of protecting the public from the loss of their health and money by the use of a dangerous nostrum.—*Druggists Circular.*

